# Salivary markers of stress system activation and social withdrawal in humans

**DOI:** 10.1192/j.eurpsy.2021.914

**Published:** 2021-08-13

**Authors:** S. Bauduin, E. Giltay, M. Van Noorden, S. Van Der Werff, M. Leeuw, A. Van Hemert, N. Van Der Wee

**Affiliations:** Department Of Psychiatry, Leiden University Medical Center, Leiden, Netherlands

**Keywords:** salivary alpha-amylase, salivary cortisol, social withdrawal

## Abstract

**Introduction:**

Social withdrawal is an early and common feature of psychiatric disorders. Hypothalamic-pituitary-adrenal (HPA)-axis activation through increased salivary cortisol (sC) and sympathetic activation through increased salivary alpha-amylase (sAA) may play a role.

**Objectives:**

We aimed to study whether the link between increased sC and sAA on the one hand and depression on the other hand is mediated by social withdrawal.

**Methods:**

In this cross-sectional, observational study, sC and sAA measures were measured in seven saliva samples in 843 participants (231 psychiatric patients and 612 healthy controls). Social withdrawal was assessed through the Brief Symptom Inventory (BSI)-, the Short Form 36-, and the Dutch Dimensional Assessment of Personality Pathology social withdrawal subscales, and analyzed using linear regression and mediation analyses. On average, participants were 44.0 years old (SD=12.8; 64.1% female).

**Results:**

Basal and diurnal sAA were unrelated to any social withdrawal scale and depression. Certain sC measures were positively associated with the BSI social withdrawal subscale (i.e., area under the curve with respect to the increase, beta=0.082, p=0.02; evening sC value: beta=0.110, p=0.003; and mean sC value: beta=0.097; p=0.01). We found limited support for statistical mediation by social withdrawal (measured using a composite social withdrawal score) on the relationship between evening sC and depression.

**Conclusions:**

Thus, although we found no support for a role of basal and diurnal sAA in social withdrawal, HPA-axis activation may partly aggravate social withdrawal in depressive disorders.

